# Fueling the engine and releasing the break: combinational therapy of cancer vaccines and immune checkpoint inhibitors

**DOI:** 10.7497/j.issn.2095-3941.2015.0046

**Published:** 2015-09

**Authors:** Jennifer Kleponis, Richard Skelton, Lei Zheng

**Affiliations:** ^1^Department of Oncology, Department of Surgery, The Sidney Kimmel Comprehensive Cancer, The Skip Viragh Center for Pancreatic Cancer Research and Clinical Care, Johns Hopkins University School of Medicine, Baltimore, Maryland 21287, USA; ^2^Masters of Health Science Program in Molecular Microbiology and Immunology, Johns Hopkins University Bloomberg School of Public Health, Baltimore, Maryland 21287, USA

**Keywords:** Cancer vaccine, immune checkpoint, immunotherapy, cytotoxic T-lymphocyte antigen-4 (CTLA-4), programmed death-1 (PD-1), programmed cell death ligand-1 (PD-L1)

## Abstract

Immune checkpoint inhibitors are increasingly drawing much attention in the therapeutic development for cancer treatment. However, many cancer patients do not respond to treatments with immune checkpoint inhibitors, partly because of the lack of tumor-infiltrating effector T cells. Cancer vaccines may prime patients for treatments with immune checkpoint inhibitors by inducing effector T-cell infiltration into the tumors and immune checkpoint signals. The combination of cancer vaccine and an immune checkpoint inhibitor may function synergistically to induce more effective antitumor immune responses, and clinical trials to test the combination are currently ongoing.

## Introduction

In recent years, the field of cancer immunotherapy development has considerably expanded with several new treatment options. This field has developed a wide array of therapies associated with the concept of immunotherapy. These therapies include cancer vaccines, adoptive cell transfer, chimeric-antigen receptor (CAR) T-cell therapy, immune checkpoint inhibitors, monoclonal antibodies, and immune system boosting techniques using interleukins. The FDA recently approved a number of novel immunotherapy agents, including checkpoint inhibitors and cancer vaccines. Sipuleucel-T, an immune-response-inducing vaccine approved in 2010, uses tumor antigens to treat prostate cancer and is the first cancer vaccine to be approved for cancer treatment. Sipuleucel-T prolonged the median overall survival of castration-resistant prostate cancer to 25.8 months compared with 21.7 months in the placebo group^1^. Among the checkpoint inhibitors, ipilimumab was approved by the FDA to treat metastatic and non-resectable melanomas in 2011^2^. In 2014, pembrolizumab and nivolumab were also approved to cure BRAF-wild-type melanoma, following ipilimumab treatment, as well as treat BRAF-mutant patients who have progressed after treatment with ipilimumab and a BRAF inhibitor^3^^,^^4^. In 2015, nivolumab was approved for chemotherapy-refractory squamous-cell type non-small cell lung cancer^5^. The recent FDA approval of various immunotherapy agents has elicited significant interest into using immune checkpoint inhibitors to target a variety of cancers. Clinical trials that have led to the FDA approval of checkpoint inhibitors all showed approximately 10% to 30% objective response rates in the approved types of malignancies at the disease stages responding minimally to previous standard treatments^2^^-^^5^.

Immune checkpoint inhibitors have contributed substantial progress to cancer treatment. However, many challenges still limit the further development of immunotherapy drugs because only about 10% to 50% of cancer patients with certain types of solid tumors have shown responses to treatments with immune checkpoint inhibitors. Such challenges are attributed to tumor microenvironment (TME) properties.

## Immune checkpoints and their clinical inhibitors

Immune checkpoint pathways have gained attention as a source of potential immunotherapeutic targets. The best studied immune checkpoint molecules include cytotoxic T-lymphocyte antigen-4 (CTLA-4), programmed death-1 (PD-1) and programmed cell death ligand-1 (PD-L1), and lymphocyte-activation gene 3 (LAG3)^6^. Through these pathways, the TME induces immune-tolerant conditions, which pose a challenge to the induction of antitumor immune responses.

The CTLA-4 pathway manipulates co-stimulatory molecule CD28 to provide a checkpoint for T-cell activation^7^. The binding of CD28 to B7.1/2 receptor serves as the second stimulation signal during the activation of T cells via the T-cell receptor. However, CTLA-4 receptors bind to B7.1/2 more strongly than CD28, resulting in inhibitory signaling^8^^,^^9^. This phenomenon induces a tolerant T-cell population within the TME, resulting in an impaired antitumor immune response^7^. Immunotherapeutic agents, such as anti-CTLA-4 antibodies, prevent the binding of CTLA-4 to the B7.1/2 receptor^10^. Ipilimumab, a monoclonal antibody targeting CTLA-4, recently received approval from the US FDA to treat metastatic melanoma. This antibody has been proven successful in phase III clinical trials for unresectable advanced stage melanoma with improved median overall survival (10.0 months) compared with the gp100 vaccine (6.4 months)^2^. In the aforementioned study, a 28.5% disease control rate and a 10.9% objective response rate in the ipilimumab groups were compared with an 11.0% disease control rate, and only 2 out of the 136 patients showed partial response in the gp100 group^2^. Clinical trials on ipilimumab treatment have been conducted to treat other cancers^11^, such as non-small cell lung cancer^12^, renal cell carcinoma^13^, and pancreatic ductal adenocarcinoma^14^. In these clinical trials, ipilimumab treatment resulted in objective responses in several cancer types, including non-small-cell lung cancer and renal cell carcinoma, but not in pancreatic ductal adenocarcinoma^12^^-^^14^.

The PD-1/PD-L1 pathway also plays a major role in the development of a tolerant TME. PD-L1 on the surface of tumor cells, antigen-presenting cells (APCs), and stromal cells are bound to the PD-1 surface molecule on T cells^15^. This binding of PD-1 and PD-L1 initiates T-cell anergy or death, thereby reducing the presence of activated effector T cells^16^. Under normal conditions, this pathway is thought to serve as a negative feedback mechanism to control the immune system following a robust inflammatory response. With regard to cancer, PD-L1 expression in tumor cells is up-regulated due to the presence of proinflammatory cytokines, such as IFN-γ, resulting in the creation of a tolerant TME^17^. In the TME, if the early influx of CD8^+^ T cells fail to clear the tumor, the tumor cells expressing high levels of PD-L1 in response to inflammation will induce T-cell anergy and lead to decreased effector T-cell activity^16^. Therapeutic blockade of this pathway is anticipated to allow for reactivation of the effector T cells in the tumor.

Anti-PD-1/PD-L1 therapies target either PD-1 or PD-L1 to prevent the binding of the receptor to its ligand as this binding leads to inactivation or anergy of CD8^+^ cells within the TME^15^. Recent FDA-approved anti-PD-1 therapeutic antibodies targeting this pathway include nivolumab and pembrolizumab^3^^,^^4^^,^^18^. Pembrolizumab and nivolumab have been demonstrated in clinical trials to improve overall survival, progression free survival, and durable response in metastatic melanoma^3^^,^^4^^,^^19^. Pembrolizumab treatment for advanced melanoma increased the 6-month progression-free survival rate (pembrolizumab every 2 weeks: 47.3% *vs*. pembrolizumab every 3 weeks: 46.4% *vs*. ipilimumab: 26.5%, *P*<0.001), as well as estimated a 12-month overall survival rate (pembrolizumab every 2 weeks: 74.1% *vs*. pembrolizumab every 3 weeks: 68.44% *vs*. ipilimumab: 58.2%, *P*=0.0036), compared with the use of the CTLA-4 checkpoint inhibitor, ipilimumab, with less adverse effects (pembrolizumab every 2 weeks: 13.3% *vs*. pembrolizumab every 3 weeks: 10.1% *vs*. ipilimumab: 19.9%)^20^. In the treatment of squamous-cell and non-small-cell lung cancers, nivolumab also improved the median overall survival compared with docetaxel (nivolumab 9.2 months *vs*. docetaxel 6.0 months, *P*<0.001)^5^. Clinical trials for anti-PD-1/PD-L1 have been conducted to treat various other cancers, such as lung adenocarcinoma^18^^,^^21^, mismatch-repair-deficient colorectal carcinoma^18^^,^^22^, renal cell carcinoma^18^^,^^23^, and bladder cancer^24^.

PD-L1/PD-1-associated checkpoint molecules, including T-LAG3, B and T lymphocyte attenuator (BTLA), T-cell membrane protein 3 (TIM3), and indoleamine 2,3-dioxygenase 1 (IDO1), are also profoundly studied as potential therapeutic targets^25^. PD-1 and LAG3 are commonly co-expressed on anergic or exhausted T cells^26^^,^^27^. Loss of LAG3 and PD-1 signaling in *Pd1^−/−^Lag3^−/−^* double-knockout mice resulted in complete rejection of poorly immunogenic tumor in a T-cell-dependent manner, and rejection in the double-knockout mice occurred much more quickly than that in *Pd1^−/−^* or *Lag3^−/−^* single-knockout mice, suggesting that these two inhibitory pathways can cooperatively suppress antitumor T effector cells^26^^,^^27^. TIM3 has also been reported to be co-expressed with PD-1 on tumor-specific CD8^+^ T cells, and the dual blockade of PD-1 and TIM3 has significantly enhanced the *in vitro* proliferation and cytokine production of T cells isolated from human melanoma patients, following stimulation with the cancer–testes antigen, NY-ESO-1^28^^-^^30^. In animal models, the combined blockade of PD1 and TIM3 has enhanced antitumor immune responses and tumor rejection compared with the blockade of PD-1 or TIM3 alone^28^^-^^30^. Additionally, CTLA-4 and PD-1 represent two T-cell-inhibitory pathways with independent mechanisms of action. CTLA-4 governs an activation threshold during the T-cell priming process. By contrast, PD-1 leads to the T-cell exhaustion limiting T-cell effector function within a tumor. Preclinical data supported the synergistic effect of dual blockade of CTLA-4 and PD-1^31^^-^^33^. The dual blockade of BTLA and PD-1 has also enhanced antitumor immunity in mouse models^25^. Supported by these preclinical data, a phase I study is being conducted for anti-LAG-3 monoclonal antibody (BMS-986016) administered alone and in combination with anti-PD-1 monoclonal antibody (nivolumab, BMS-936558) in advanced solid tumors (NCT01968109). Anti-Tim-3 therapeutic antibodies have been developed and are awaiting phase I testing. Other B7 family members of co-inhibitory molecules are also being targeted. Phase I studies of anti-B7-H3 antibodies (MGA271) alone or in combination with ipilimumab in refractory cancer are ongoing (NCT01391143; NCT02381314). Multiple IDO inhibitors have been developed. Phase I studies of indoximod have shown the safety of this IDO inhibitor and the potential of its efficacy^34^. These new agents are expected to further enhance the antitumor response to the anti-PD-1 antibody and anti-CTLA-4 antibody treatments. However, whether they are administered alone or in combination with other checkpoint inhibitors to overcome the resistance toward anti-PD-1 and anti-CTLA-4 antibodies in “non-immunogenic” cancers remains to be tested.

Clinical studies have already investigated the combinational therapy of anti-PD-1/PD-L1 therapies together with other checkpoint inhibitors, such as anti-CTLA4 treatments with ipilimumab^35^^,^^36^. The combination of nivolumab and ipilimumab increased the rate and degree of tumor regression (53% with objective responses and tumor reduction of 80% or more) compared with single-checkpoint-inhibitor treatment (20% to 30% with objective responses) in clinical trials to treat advanced melanoma^35^. High-grade immune-related adverse events (irAEs) occurred in 53% of the patients who received ipilimumab and nivolumab concurrently^35^, and this rate was higher than those observed with single-checkpoint-inhibitor treatments^3^^,^^4^^,^^19^.

## Immune checkpoint inhibitors function on T cells

**Figure 1:**
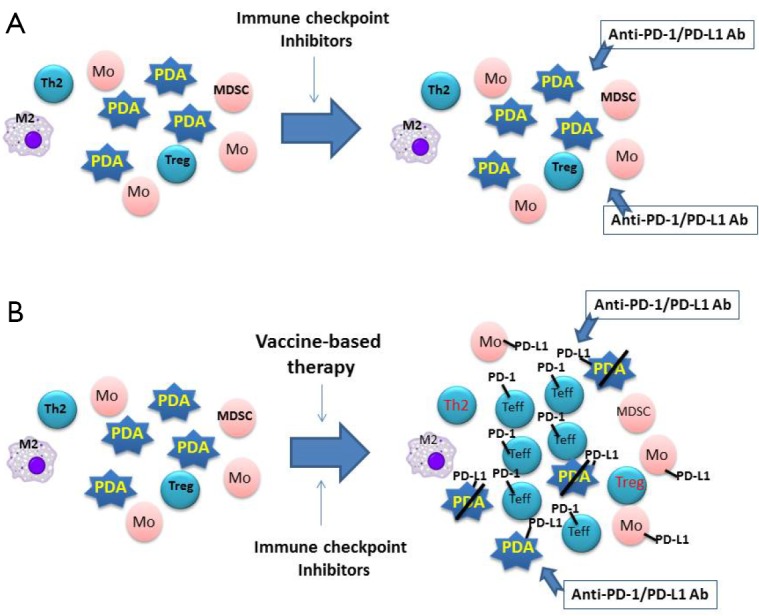
Model for the combination of vaccine-based therapy and immune checkpoint inhibitors. (A) Pancreatic ductal adenocarcinoma (PDA) is infiltrated primarily with M2 macrophages (M2), type 2 T helper cells (Th2), myeloid-derived suppressive cells (MDSC), and regulatory T cells (Treg) but with few effector T cells (Teffs). Lacking PD-1/PD-L1 targets, PDA does not respond to single-agent checkpoint inhibitor treatments, such as anti-PD-1 or PD-L1 therapeutic antibodies (anti-PD-1/PD-L1 Ab). (B) Following vaccine-based therapy, vaccine-induced Teffs are infiltrated into PDA; however, PD-L1/PD-L1-mediated immune checkpoint pathways are also induced. By targeting PD-L1/PD-L1 signals on PDA tumor cells and monocytes (Mo) induced by vaccine-based therapy, anti-PD-1/PD-L1 therapeutic antibodies enhance vaccine-induced antitumor immune responses.

Generally, T cells are the primary target of the above described therapeutic immune checkpoint inhibitors, as well as those in development. Effector T-cell infiltration in solid tumors appears to be a signature trait of patients who responded to treatment with immune checkpoint inhibitors^37^^,^^38^. This signature characteristic has determined that only a fraction of solid tumor patients respond to the immune checkpoint inhibitors. The fraction of patients who responded to these treatments include 20% to 50% of melanoma patients^18^^,^^35^^,^^36^, 20% to 30% of non-small-cell lung cancer patients^5^^,^^18^^,^^39^, 20% to 30% of renal cell carcinoma patients^18^^,^^39^, and 10% to 20% of colorectal cancer patients with a mismatched-repair deficiency^18^^,^^22^. The remaining cancer patients would unlikely respond to the immune checkpoint inhibitors as single-agent treatments because of the lack of targets. Tumors in these patients are naturally depleted by effector immune cells, resulting in a reduction of checkpoint targets for immunotherapy^40^^-^^44^. One example of this phenomenon is pancreatic cancer, which features a highly tolerant, “immune quiescent” TME^40^^,^^41^. Effector T cells may have been exhausted by the chronic inflammatory process associated with tumorigenesis, but this process is not strong enough to reject the malignantly transformed cells^45^. In “immune quiescent” tumors, such as pancreatic carcinoma, PD-L1 expression is also low^46^. Objective responses have not been reported with pancreatic cancer cases treated by single-agent checkpoint inhibitors^39^^,^^47^. The TME in these immune checkpoint inhibitor-resistant tumors is similar to an engine without gas. Even if the “brake” set by immune checkpoints is released through immune checkpoint inhibitor immunotherapy, no effective antitumor immune response would be elicited (**Figure 1**).

## Vaccines: fueling the TME with T Cells

Cancer vaccines have been shown to enhance effector T-cell infiltration into the tumors in preclinical models. The major types of cancer vaccines include peptide vaccines, vector-based antigen specific vaccines, whole-cell vaccines, and dendritic cell vaccines^48^. All vaccine-based therapies are designed to deliver either single or multiple antigenic epitopes or antigens from the whole cells to the patients and induce tumor-specific effector T cells. Thus, a vaccine-based therapy may be the most efficient way to induce T-cell infiltration into the tumor. However, whether the vaccine-induced immune response would actually take effect on the TME is debatable.

Our group at the Johns Hopkins University developed the granulocyte macrophage colony-stimulating factor (GM-CSF)-secreting pancreatic cancer vaccine (GVAX)^49^^-^^51^. The use of whole-cell vaccines is promising because it delivers a range of antigens without the need for specific knowledge of the relevant target antigens. Pancreatic GVAX consists of two allogeneic pancreatic tumor cell lines that have been modified with a plasmid vector encoding the cDNA for human GM-CSF^49^. The GM-CSF simultaneously recruits and provides maturation signals to APCs to the local vaccine site. The recruited APCs then orchestrate an immune response by processing tumor antigens expressed by the vaccine PDA cell lines and presenting them to the patient’s T effector cells. Studies evaluating GVAX in patients with both resected and metastatic PDA have shown that GVAX induces enhanced T-cell responses specific to mesothelin, an antigen expressed commonly by PDAs and also by GVAX, in a subset of patients associated with longer survival^50^^-^^52^.

Our group recently completed a neo-adjuvant and adjuvant research designed to evaluate post-immunotherapy changes within the TME of primary pancreatic tumors following treatment with this vaccine. The vaccine was given either alone or with immune modulating doses of cyclophosphamide to deplete regulatory T cells. Pathological examination of tumor tissue resected only 2 weeks following vaccination identified the formation of novel immunotherapy-induced lymphoid aggregates. These organized tertiary lymphoid structures are not observed in tumors resected from unvaccinated patients. This study showed for the first time that treatment with a vaccine-based immunotherapy directly alters the pancreatic cancer TME, allowing infiltration of organized and functional immune structures that convert an immunologically quiescent tumor into an immunologically active tumor^46^.

The above study also demonstrated that the formation of these immune regulatory structures within the TME is only the first step toward establishing an enhanced anticancer immune response, which is attributed to the ability of these lymphoid aggregates to express both effector-activating and effector down-regulating immune signatures. Interestingly, PD-L1 expression was induced in all these lymphoid aggregates^43^. This observation is consistent with the presence of adaptive immune resistance when the PD-L1 signaling is activated by vaccine-induced adaptive immune response^25^. Thus, vaccine-based therapies may have primed pancreatic cancer for anti-PD-1/PD-L1 treatments^46^^,^^53^.

## Fueling the engine and releasing the break: combination therapy

Conceivably, the combination of vaccine therapy and immune checkpoint inhibitors may synergistically induce antitumor immune responses. This notion has been supported by studies with preclinical models. First, Karyampudi *et al*.^54^ demonstrated that an anti-PD-1 antibody and a multi-peptide vaccine consisting of immunogenic peptides derived from breast cancer antigens, neu, legumain, and β-catenin served as a combination therapy regimen, which prolonged the vaccine-induced progression-free survival of breast tumor-bearing mice. Second, Li *et al*.^55^ and Soares *et al*.^44^ showed that anti-PD-1/PD-L1 antibodies enhanced antitumor activities of the GM-CSF-secreting cancer vaccine (GVAX) in both mouse models of colon cancer and pancreatic cancer, respectively. Third, Fu *et al*.^56^ showed that cyclic dinucleotides formulated GVAX (termed “STINGVAX”), which demonstrated potent *in vivo* antitumor efficacy in multiple preclinical models of established cancer. Combined with anti-PD-1 blockade antibodies, STINGVAX induced regression of tumors that did not respond to PD-1 blockade alone. Fourth, Curran *et al*.^31^ and Duraiswamy *et al*.^32^ showed that dual blockade of PD-1 and CTLA-4 combined with vaccines more effectively eradicated tumors in multiple preclinical models.

Supported by the above preclinical data^44^, a clinical trial to test the pancreatic cancer vaccine-based therapy in combination with nivolumab for metastatic pancreatic cancer has been initiated (ClinicalTrials.gov identifier: NCT02243371). Furthermore, a novel clinical trial to test the combination of GVAX and nivolumab as neoadjuvant and adjuvant therapies for resectable pancreatic cancer will be initiated (ClinicalTrials.gov identifier: NCT02451982).

Whether anti-PD-1 therapeutic antibodies can effectively enhance the efficacy of cancer vaccines in treating pancreatic cancer remains to be investigated. The combination of GVAX and anti-CTLA-4 antibody, ipilimumab, has also shown to be potentially effective in treating metastatic pancreatic cancer. In a randomized study of metastatic pancreatic cancer patients who have been resistant to multiple lines of chemotherapy, the combination of GVAX and ipilimumab led to objective responses in 3 out of 15 patients, whereas no objective response was observed with any of the 15 patients treated with ipilimumab alone^47^. The objective response rate of 20% with the combination of GVAX and ipilimumab approximated that either anti-CTLA-4 antibody or anti-PD-1 antibody alone in treated non-small-cell lung cancer, renal cell carcinoma, gastric adenocarcinoma, and hepatocellular carcinoma. Strong response was observed in one patient who initially received GVAX as a participant in the abovementioned neoadjuvant and adjuvant vaccine research^43^. After this patient presented a recurrence, he received additional chemotherapy and radiation therapy but continued to exhibit disease progression. Later, when we analyzed his tumor together with the other PDA tumors from the neoadjuvant and adjuvant vaccine research, we found that the lymphoid aggregates formed in his surgically resected PDA showed an immune suppressive signature, which was characterized by a relatively high density of Foxp3^+^ cells, albeit high density of CD8^+^ cells and relatively high expression of CTLA-4. After he had received the combination of ipilimumab and GVAX treatments, he demonstrated an early local progression and developed a new omental lesion at week 7 after beginning the combination treatment but followed by strong disease stabilization starting at week 22^47^. At 5 years after recurrence, this patient remains alive and is 3 years out from his last treatment. Although his CT scan still showed soft-tissue density in the local pancreatic region and peritoneal nodularity, biopsy of these lesions failed to demonstrate malignant cells. These data, albeit anecdotal, suggest that the combination of checkpoint inhibitors and vaccine therapies may reverse an unfavorable TME dominated by immune suppressive signals and allowing the generation of a productive antitumor response.

Nevertheless, although GVAX was found in the above study to only add the toxicity profile with self-limited regional or systemic rashes, ipilimumab was associated with frequent irAEs^47^. Up to 73% of patients in the ipilimumab arm and 80% in the ipilimumab/GVAX combinational arm experienced any grade irAE, and 20% of the patients in both arms experienced grade 3 and 4 irAEs (colitis, Guillain-Barre syndrome, nephritis, rash, and pneumonitis). Therefore, anti-PD-1 blockade antibodies, which exhibit a low autoimmune toxicity profile, may serve as better candidates to combine with vaccine-based therapies. Whether the combination of anti-PD-1 antibodies and GVAX will result in objective responses and prolonged survival remains to be tested in the aforementioned clinical trials. The neoadjuvant study of the GVAX/nivolumab combination will provide an opportunity to identify other immune checkpoint or activation pathways that may further enhance the antitumor immune response. Combining vaccine therapy with dual blockade of CTLA-4 and PD-1 might be interesting, although the autoimmune toxicities can be a concern. However, the combination of vaccine and PD-1/PD-L1 blockade may be further combined with checkpoint inhibitors with modest toxicities, targeted therapies, or radiation therapies to achieve synergistic antitumor activities.

In summary, cancer vaccine-based immunotherapy may overcome the resistance of certain cancers to immune checkpoint inhibitors, while immune checkpoint inhibitors may enhance the efficacy of the cancer-vaccine therapies (**Figure 1**). The strength of a combination immunotherapy combines the strength of each immunotherapy approach, with cancer vaccine to fuel the engine, and with immune checkpoint inhibitor to release the brake.
